# No detection of CD4-independent human immunodeficiency virus 1 envelope glycoproteins in brain tissue of patients with or without neurological complications

**DOI:** 10.1007/s00705-018-4094-1

**Published:** 2018-11-10

**Authors:** Briana Quitadamo, Paul J. Peters, Matthew Koch, Katherine Luzuriaga, Cecilia Cheng-Mayer, Paul R. Clapham, Maria Paz Gonzalez-Perez

**Affiliations:** 1 Biotech 2, Program in Molecular Medicine, University of Massachusetts Medical School, Suite 315, 373 Plantation Street, Worcester, MA 01605 USA; 2 Biotech 2, University of Massachusetts Medical School, Suite 318, 373 Plantation Street, Worcester, MA 01605 USA; 3 The Aaron Diamond AIDS Research Center, 455 First Avenue, 7th Floor, New York, NY 10016 USA

## Abstract

Macrophage (mac)-tropic human immnunodeficiency virus type 1 (HIV-1) and simian immnunodeficiency virus (SIV) in brain are associated with neurological disease. Mac-tropic HIV-1 evolves enhanced CD4 interactions that enable macrophage infection via CD4, which is in low abundance. In contrast, mac-tropic SIV is associated with CD4-independent infection via direct CCR5 binding. Recently, mac-tropic simian-human immunodeficiency virus (SHIV) from macaque brain was also reported to infect cells via CCR5 without CD4. Since SHIV envelope proteins (Envs) are derived from HIV-1, we tested more than 100 HIV-1 clade B Envs for infection of CD4-negative, CCR5^+^ Cf2Th/CCR5 cells. However, no infection was detected. Our data suggest that there are differences in the evolution of mac-tropism in SIV and SHIV compared to HIV-1 clade B due to enhanced interactions with CCR5 and CD4, respectively.

## Introduction

HIV-1, HIV-2 and SIV each infect brain tissue and can cause neurological complications including encephalopathy and dementia [6, 36, 59]. Overall, about 7% of untreated HIV-1^+^ patients (30% of AIDS patients) suffer from severe HIV-associated dementia (HAD) [6, 22]. Since the introduction of combination antiretroviral therapy, HAD and other neurocognitive complications in HIV-1^+^ subjects have been greatly reduced, with only 1% of patients suffering from HAD [6].

HIV-1 colonizes the brain during the acute stage of infection [11]. However, proviral DNA is difficult to detect in brain tissue during the asymptomatic phase [3, 12, 34]. Perivascular macrophages are the main cell target for HIV-1 in the brain [16, 17, 19, 22, 29, 52, 60], with resident microglia (macrophage lineage) also becoming infected [9, 16, 17]. Some studies have indicated that astrocytes that do not express CD4 can also be infected [1, 7, 44, 52], particularly in pediatric cases [47, 50, 56].

HIV-1 infection of cells is driven by interaction of the envelope glycoprotein (Env) with the CD4 receptor and a coreceptor, usually CCR5 [58]. The vast majority of HIV-1 variants outside the central nervous system do not efficiently infect macrophages and replicate in CD4^+^ T cells. These CCR5-using variants have been termed “R5 non-macrophage-tropic” [40] or “R5 T-cell tropic” [23, 49]. Colonization of the brain by HIV-1 requires the emergence of R5 macrophage (mac)-tropic variants that efficiently infect macrophages or microglia present there [24, 39, 40] and are almost universally found in brain tissue of patients with severe neurological complications. These variants are adapted to exploit the substantially lower levels of CD4 on the surface of macrophages compared to T cells [15, 27, 41, 43] and interact with CD4 via a high affinity Env:CD4 interaction [43].

Several SIV/macaque animal models of neuropathogenesis have been developed [59], and a number of SIV variants from brain tissue of these models have been described [59]. Similar to HIV-1, these variants from brain tissue are highly mac-tropic [18, 61]. Such SIV variants are, however, frequently CD4-independent and are able to infect CD4-negative cell lines via a direct interaction with the CCR5 coreceptor [42]. Moreover, a more recent study of simian-human immunodeficiency virus (SHIV) infection of macaques also described the presence of CD4-independent Env variants present in brain tissue [62]. These models of neurological disease often require immune suppression/modulation as well as adaptation to use macaque CD4 [4, 59, 62]. Since SHIVs carry env genes derived from HIV-1, this observation suggested that CD4 independence may be a general or frequent property of HIV/SIV Envs and variants present in brain tissue.

Previously, in our early studies using relatively limited numbers of mac-tropic HIV-1 Envs from brain tissue, we did not identify any HIV-1 Envs with the capacity to infect CCR5^+^ cells in the absence of CD4 [39]. Here, we have tested a large number of HIV-1 subtype B Envs derived by single-genome PCR from brain and other tissues of subjects with and without neuroAIDS. We have also investigated variation in Env:CCR5 interactions that might indicate an adaptation for enhanced Env:CCR5 interactions required for CD4 independence.

## Materials and methods

### Patient tissue samples

Envelope genes were derived from the patients listed in Table 1. Full patient details have been published previously [21].

**Table 1:** HIV-1^+^ patients investigated

Patient no.	Brain bank or origin	Neurological disease status^a^	Years of infection	CD4 count	Viral load
				Cells/mm^3^	Plasma
CA110	UCSD	NeuroAIDS (0)^b^	21	21 (60)	198,957 (60)
7766	Texas	NeuroAIDS (0)^b^	12	43 (21)	1,843 (20)
6568	Texas	NeuroAIDS (163)	17	77 (79)	>750,000 (402)
10017	Mt. Sinai	NeuroAIDS (191)	9	7 (319)	389,120 (381)
NA20	Edinburgh	NeuroAIDS	nd^c^	4	nd
NA420	Edinburgh	NeuroAIDS	nd	12	nd
JR	UCLA	NeuroAIDS	nd	nd	nd
P1114	UMMS^d^	NeuroAIDS	nd	46	nd
CE161	UCSD	Normal^e^ (192)	13	11 (192)	246,000 (192)
8276	Texas	Normal (146)	6	3 (182)	750,000 (184)
6771	Texas	Normal (310)	10	23 (354)	75,000 (37)
5057	UCLA	Normal (253)	12	663 (148)	29,282 (-1)
6052	UCLA	Normal (24)	14	13 (24)	75,000 (24)

^a^Bracketed values represent the number of days before death that assessments or measurements were made

^b^Diagnosis made from patient information after death

^c^*nd* no data

^d^UMMS, University of Massachusetts Medical School

^e^Normal neurocognitive diagnoses

### Cell cultures

Env^+^ pseudovirions were prepared in 293T cells by transfection. HeLa TZM-bl cells [57] were used to estimate Env^+^ pseudovirion infectivity titers. HeLa TZM-bl cells express high levels of CD4, CCR5 and CXCR4 and contain HIV-inducible β-galactosidase and luciferase reporter genes. Canine thymus epithelial cells expressing human CCR5 (Cf2Th/CCR5 cells) were used to evaluate HIV-1 and SHIV CD4-independent infection via CCR5 [62]. 293T cells, TZM-bl cells, and Cf2Th/CCR5 cells were maintained in Dulbecco’s modified Eagle’s medium (DMEM, Gibco-Invitrogen, Carlsbad, CA) supplemented with 5% fetal bovine serum (FBS).

Macrophage cultures were prepared from blood monocytes by adherence using buffy coats provided by New York Biologics Inv. as described previously [13, 14, 39]. Monocytes were cultured for 5 days in DMEM medium containing 10% AB+ male human serum (HS) for differentiation before setting up for infection. On the day prior to infection, the macrophages were washed and resuspended in DMEM containing 10% HS and cultured in 48-well tissue culture plates (1.25×10^5^ cells/well; 0.5 ml/well).

### Env^+^ pseudotype assays

Env^+^ pseudovirions were produced as described previously [39]. Briefly, pseudoviruses were prepared by cotransfection of 293T cells with env^+^ pTOPOenv vector and an env-minus pNL4.3Δenv using a calcium phosphate transfection kit (Profection; Promega Inc.). Cell-free supernatants were harvested after culturing for 48 h, and aliquots were frozen at -152 °C prior to analysis.

Env^+^ pseudovirions were titrated on HeLa TZM-bl cells, Cf2Th/CCR5 cells, and primary macrophages [39]. For HeLa TZM-bl and Cf2Th/CCR5 cells, 2×10^4^ cells in 0.5 ml were added to each well of 48 well plates the day prior to virus titration. One hundred µl of serially diluted viral supernatants in DMEM medium containing 5% FBS was added to the cells, which were then incubated for 3 h. Then, 0.4 ml of DMEM containing 5% FBS was added and the cultures were incubated for 48 h. (Hela TZM-bl) or 72 h. (Cf2Th/CCR5). TZM-bl cells were fixed in 0.5% gluteraldehyde in PBS and stained for β-galactosidase expression using X-gal substrate (0.5 mg of X-gal per ml, 3 mM potassium ferricyanide, 3 mM potassium ferrocyanide, 1 mM magnesium chloride). Cf2Th/CCR5 cells were fixed in cold methanol:acetone (1:1), washed, and immunostained for p24 using monoclonal antibodies 38:96 K and EF7 (UK Centre for AIDS Research), followed by an anti-mouse IgG-β-galactosidase conjugate and X-gal substrate [39].

Macrophages were seeded in 48-well plates and pretreated with 0.1 ml of DEAE dextran (10 µg/ml) in DMEM medium containing 10% HS for 30 min at 37 °C. The virus supernatants were then added, and the plates inoculated by spinoculation for 45 minutes in a benchtop centrifuge [38]. Infected macrophages were incubated for a further 3 h at 37 °C before the addition of 0.4 ml of DMEM (10% AB^+^ male HS) and incubation at 37 °C for seven days. Macrophages were then fixed and immunostained for p24 as described for Cf2Th/CCR5 cells. DEAE dextran and spinoculation enhance virus infectivity by up to 20-fold by increasing attachment [38] and entry [26]. Infection following this procedure helps to maximize macrophage infection and allows the most mac-tropic Env^+^ pseudoviruses to be distinguished. It does not bypass the requirement of CD4 and CCR5 for infection, and the system remains sensitive to entry inhibitors, including maraviroc (not shown). Env^+^ pseudovirion titers were expressed as focus-forming units (FFU) estimated by counting individual or small groups of blue-stained infected cells by light microscopy.

All titration values for Cf2Th/CCR5 cells, HeLa TZM-bl cells, and macrophages represent averages of at least two independent experiments, each done in duplicate. In addition, the macrophage experiments were repeated using cells from different donors. Error bars in figures were calculated from replicate wells of both experiments.

### Soluble gp120 production and gp120:CCR5 binding assays

Soluble gp120 was produced in 293F cells. 1 × 10^6^ cells/ml in 500 ml were transfected with 250 μg DNA of pJW4303 containing different gp120s [31] using a suspension of 293Fectin (Invitrogen Inc.) following the manufacturer’s instructions. Transfected cells were cultured in Freestyle 293 Expression Medium (Invitrogen Inc.) in shaking flasks for 72 hours. Cells were then pelleted by centrifugation, supernatants were harvested, gp120 was purified using a lectin column. Then, gp120s were concentrated using 50 K-MW-cutoff centrifugal filter concentrators (Millipore Inc.).

Soluble gp120 binding to CCR5 on the surface of Cf2Th/CCR5 cells was measured by flow cytometry. Cf2Th/CCR5 cells were first detached from culture plates using versene. One μg of gp120 in 100 μl of PBS containing 1% FBS was incubated at room temperature for 30 minutes in the absence or presence of 0.5 μg of sCD4 in 96-well V-bottom plates. One hundred μl containing 5 × 10^6^ Cf2Th/CCR5 cells was then added, and the plates were incubated for 60 min at room temperature. Cells were washed with PBS containing 1% FBS, and attached gp120 was detected by treating with HIV^+^ serum mix (1:2500) and incubation for 30 minutes at 4 °C before washing again and treating with anti-human IgG-FITC conjugate (Southern Biotech Inc.) at 4 °C for 30 min. Cells were then washed again in PBS plus 1% FBS, followed by PBS alone, and were fixed in 4% formaldehyde in PBS. Fixed cells from each well were then filtered through a 35-μm filter capped tube (Falcon Inc.) and analyzed by flow cytometry at the UMASS Medical School Flow Cytometry Core Lab using a BD LSRII cytometer and DIVA 8.0 acquisition software. Flow analysis was performed using Flowjo, 10.1r7. Appropriate instrument, assay and analysis controls were included in all runs.

### Phylogenetic analysis

Phylogenetic and molecular evolutionary analysis was conducted using MEGA, version 7 [53]. Maximum-likelihood phylogenetic trees were generated using the general time-reversible model with gamma distribution. Bootstrap analysis with 1,000 replicates was used to assess the robustness of the tree. Significant (≥70%) bootstrap values are shown at internal tree nodes. Reference nucleotide sequences representing four HIV-1 group M subtype B (http://www.hiv.lanl.gov/) envelope proteins (FR.83.HXBc2.K03455, TH.90.BK132.AY173951, US.98.1058_11.AY331295, and NL.00.671_00T36.AY423387) as well as SHIV mac-tropic envelope proteins (AD8.JN560961.1, and SF162P3_MC_2011.3.JQ672558.1) were used as outgroups.

### Statistics

Differences between CD4-independent infection mediated by HIV-1 and SHIV Envs were evaluated using non-parametric Mann-Whitney tests. Binding of mac-tropic and non-mac-tropic HIV-1 gp120s to CCR5 was compared using Wilcoxon matched pairs tests. Finally, significant inhibition of HIV-1 gp120:CCR5 binding using maraviroc was tested using unpaired *t*-tests.

## Results

### No detection of CD4-independent infection by HIV-1 Envs in brain and immune tissue of AIDS patients

Previously, we tested whether pseudoviruses carrying 14 Envs derived from five subjects with neurological disease infected cells expressing CCR5 but not CD4. This relatively small panel of Envs included eight highly mac-tropic Envs from brain tissue. In that earlier study, none of the Env^+^ pseudoviruses were able to infect cells in the absence of CD4 [39]. Here, we extended our study to more than 100 further Envs derived from brain or immune tissue of four neuroAIDS patients and five AIDS patients without or with only minor neurological complications (Table 1) [20, 21]. The canine thymus epithelial CF2Th cells expressing human CCR5 (Cf2Th/CCR5) were used to test CD4-independent infection since these cells were used by Zhuang et al. [62] to assess infection in the absence of CD4 by SHIV Env^+^ pseudoviruses. Control pseudoviruses included those carrying VSV G protein and SHIV Envs #3 and #7, which were reported to be CD4 independent by Zhuang et al. [62].

**Fig. 1:**
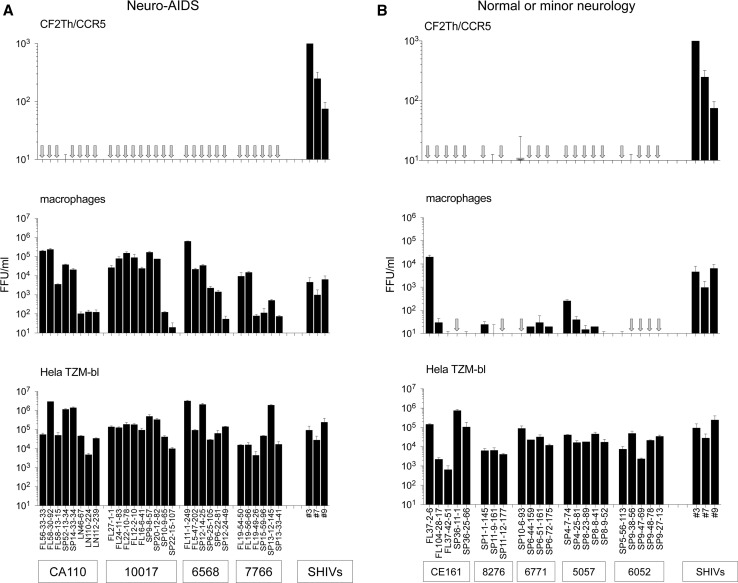
No detection of CD4-independent infection by HIV-1 Env^+^ pseudoviruses carrying Envs from brain and immune tissue of AIDS patients. (A) Pseudoviruses carrying HIV-1 Envs derived from brain frontal lobe (FL), spleen (SP), or lymph node (LN) tissues of subjects with neuroAIDS were titrated for infection of CD4-negative Cf2Th/CCR5 cells, primary macrophages, and HeLa TZM-bl cells. No significant infection of Cf2Th/CCR5 cells was detected for any Env^+^ pseudovirus tested, despite high levels of infectivity detected for macrophages and HeLa TZM-bl cells. In contrast, three different SHIV Envs derived from brain tissue of an SHIV-infected macaque mediated significant CD4-independent infection of Cf2Th/CCR5 cells as well as substantial infection of macrophages and HeLa TZM-bl cells (right 3 bars). (B). Pseudoviruses carrying HIV-1 Envs derived from brain frontal lobe or spleen tissues derived from subjects without or with only minor neurological issues failed to mediate infection of Cf2Th/CCR5 cells. Most Envs derived from these subjects were not mac-tropic despite conferred high levels of infection for HeLa TZM-bl cells

Fig. 1A shows that none of the HIV-1 Envs from neuroAIDS tissues conferred significant infection of Cf2Th/CCR5 cells. In this experiment, we included highly mac-tropic Envs from frontal lobe tissue (designated FL) of four neuroAIDS patients as well as several Envs from lymph node (LN) and spleen (SP) that we had previously shown to mediate low, modest or high mac-tropism [20]. Here, all of these Envs mediated substantial levels of infectivity for HeLa TZM-bl cells, while high levels of macrophage infection were recorded for all pseudoviruses carrying Envs derived from brain tissue and several rare examples of mac-tropic Envs from lymph node and spleen, as expected. We also tested pseudoviruses carrying three SHIV Envs (#3, #7 and #9) described by Zhuang et al. study [62]. These SHIV Envs confer high, medium, and modest infection of Cf2Th/CCR5 cells, respectively (Fig. 1A, right-hand bars), confirming the previous data. The infectivity data presented in Fig. 1A, middle panel, also confirm that these brain-derived SHIV Envs are mac-tropic.

We also tested pseudoviruses carrying HIV-1 Envs from subjects without or with only minor neurological complications for infection of CD4-negative Cf2Th/CCR5 cells. Three of these Envs were from the frontal lobe of subject CE161 (one of which, Env FL37-2-6, was a highly mac-tropic Env), and the remainder were from spleen tissue. No significant infection of Cf2Th/CCR5 cells was detected for any of the Env^+^ pseudovirus tested despite high levels of infectivity detected for HeLa TZM-bl cells (Fig. 1B).

Together, these data show that HIV-1 Envs derived from brain or immune tissue of patients with or without neuroAIDS were unable to mediate infection of cells expressing CCR5 but not CD4. Collectively, these Envs were significantly less able to mediate CD4-independent infection compared to the control SHIV Envs, which varied from low to high CD4 independence (*p* < 0.0001).

### Mac-tropic HIV-1 Envs from neuroAIDS do not evolve a high-affinity gp120:CCR5 interaction

**Fig. 2:**
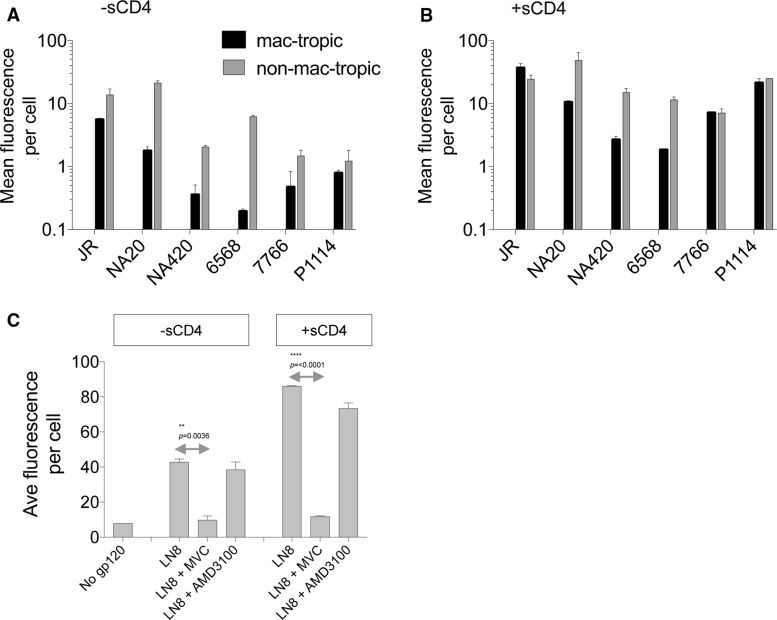
Mac-tropic and non-mac-tropic soluble gp120 binding to cell-surface CCR5. Soluble gp120s from highly mac-tropic and non-mac-tropic R5 Envs from subjects JR, NA20, NA420, 6568, 7766 and P1114 were investigated for binding to CCR5. (A and B) Soluble gp120 binding to canine Cf2Th/CCR5 cells in the absence (A) and presence of sCD4 (B). 10 μg of gp120 per ml with or without sCD4 (5 μg/ml) was bound to Cf2Th/CCR5 and detected by flow cytometry using a mixture of HIV-1^+^ human sera (1:2,500) and an anti-human Ig FITC conjugate. Soluble CD4 enhanced binding as expected (B). Soluble gp120 from non-mac-tropic Envs bound CCR5 in the absence of CD4 more efficiently than gp120s from mac-tropic Envs (*p* = 0.031), and sometimes more efficiently in the presence of sCD4 (not significant). (C) NA20 LN8 binding to CCR5 was inhibited by the CCR5-specific antagonist maraviroc, but not by the CXCR4 antagonist AMD3100, as expected

For HIV or SIV Envs to trigger entry into CCR5^+^ cells in the absence of CD4, it is likely that a high-affinity Env:CCR5 interaction would need to evolve [25]. We next evaluated whether mac-tropic R5 Envs from brain tissue of neuroAIDS patients had evolved an enhanced binding affinity for CCR5 that might facilitate CD4-independent infection via CCR5. For this experiment, soluble gp120s were prepared from a panel consisting of mac-tropic and non-mac-tropic R5 Env pairs derived from six neuroAIDS subjects, including five highly mac-tropic, brain-derived Envs (Table 1, Fig. 2). This panel included additional Envs derived from patients NA20, NA420, P1114 and JR, subjects that have been described in detail previously [39, 40]. They were among the first small group of Envs that we tested previously for CD4 independence with negative results [39].

Env binding to CCR5 was tested by measuring soluble gp120 attachment to CCR5 expressed on Cf2Th/CCR5 cells [28]. Binding was tested in the absence and presence of sCD4. Bound gp120 or gp120/sCD4 was then detected using a pool of HIV-1^+^ sera and an anti-human IgG-FITC with staining measured by flow cytometry (Materials and methods).

Binding of several non-mac-tropic and mac-tropic gp120s to CCR5 was detected in the absence of sCD4. This binding was variable, with each of the non-mac-tropic gp120s binding CCR5 significantly more efficiently (*p* = 0.031) than the mac-tropic gp120s from the same subjects (Fig. 2A).

For all gp120s tested, sCD4 greatly enhanced gp120 binding to CCR5, as expected (Fig. 2B), with non-mac-tropic gp120s maintaining higher CCR5 binding capacity compared to their counterpart mac-tropic gp120s for the three subjects studied, although this was not significant. Finally, the presence of 5 µg of maraviroc per ml blocked LN8 and LN8/sCD4 complexes from binding CCR5^+^ Cf2Th/CCR5 cells, while 5 µg of CXCR4-specific AMD3100 per ml had no effect. This last observation confirms that the binding was CCR5 specific (Fig. 2B).

In summary, gp120s derived from immune tissue of neuroAIDS subjects universally maintain strong binding for CCR5, while gp120s from brain are more variable, with several binding significantly less efficiently.

### Compartmentalization of mac-tropic R5 Envs from brain tissue of AIDS patients

**Fig. 3:**
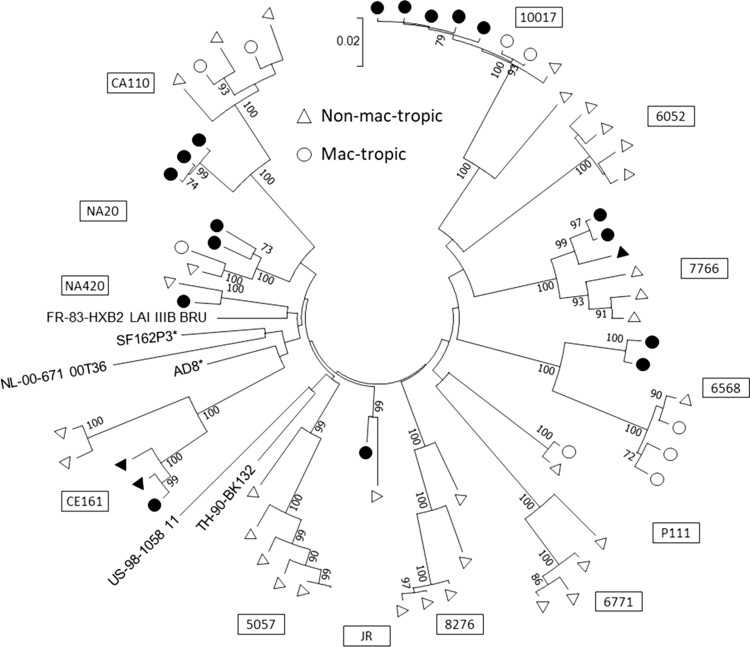
Mac-tropic R5 Envs from brain tissue of AIDS patients cluster together. A phylogenetic tree was constructed by the maximum-likelihood method, and macrophage infectivities of HIV-1 envelope nucleotide sequences from brain (closed circles) and immune tissue/CSF (open circles) of HIV^+^ individuals are shown. Numbers at branch points represent bootstrap values (≥70%)

We investigated the relationship between macrophage tropism and population diversity of *env* sequences from brain and immune tissue of 13 HIV^+^ subjects with or without neurological complications. A phylogenetic tree was plotted that included representative sequences from all individuals studied (Fig. 3). The average evolutionary divergence estimate was 0.102 (S.E. = 0.009). *Env* sequences from each individual segregated separately, which is consistent with their different origins and indicate that a diverse clade B env population was studied.

Our studies also confirm that there is strong compartmentalization of highly mac-tropic R5 HIV variants that predominate in brain tissue of individuals with neuroAIDS. In contrast, Envs from immune tissues mostly varied in mac-tropism from background to modest, but in a minority of Envs, mac-tropism was highly efficient [20]. Fewer mac-tropic Envs were present in brain tissue of patients without neurological complications and formed phylogenetic clusters distinct from those of most non-mac-tropic Envs from the immune tissue or brain [21]. More-detailed amino acid sequence analysis was published previously [20, 21].

## Discussion

Previously, we reported that CD4 independence was a property that is associated more with Envs of HIV-2 and SIV isolates than with those of HIV-1 [8, 45]. However, a more recent report from Cheng-Mayer and coworkers group described CD4-independent, mac-tropic SHIV variants that are present in brain tissue of macaques with neurological disease [62]. Since SHIV env genes are derived from HIV-1, we were motivated to investigate whether any of a large panel HIV-1 R5 Envs derived from AIDS patients could also infect CCR5-expressing cells without CD4. These Envs were derived mainly from brain and immune tissue from subjects infected with HIV-1 of clade B, and without neurological disease, as we have described previously [20, 21]. Here, no evidence of CD4-independent infection was detected for Env^+^ pseudoviruses carrying any of the more than 100 HIV-1 Envs tested.

Previous studies by our group and others have indicated that HIV-1 mac-tropism in brain tissue involves the evolution of high-affinity Env:CD4 interactions so that small amounts of CD4 on macrophages can be exploited to trigger viral entry [15, 37, 41, 43]. It is unclear whether similar changes occur in SIV or HIV-2. However, CD4 independence is indicative of an increase in the exposure for the coreceptor binding site on Env, perhaps combined with enhanced Env:CCR5 affinity. This capacity to infect coreceptor^+^ cells without CD4 will obviously help variants to infect macrophages that express only low amounts of CD4 [2, 30, 35] and could explain infection of astrocytes, which is sometimes detected in late-stage neurological disease [1, 7, 44, 47, 50, 52, 56]. Thus, it is likely that low amounts of CD4 on the cell surface of macrophages may greatly enhance infection by CD4-independent variants by augmenting virus binding or acting cooperatively to trigger conformational changes leading to fusion [8]. Together, these different observations support two evolutionary routes to mac-tropism, one involving enhanced Env:CD4 interactions for HIV-1 and the second involving enhanced Env:CCR5 interactions for SIV and SHIV.

An earlier report describing CD4-independent SHIVs (which carry HIV-1 clade-C-derived Envs) in macaque brain [62] contrasts with the presence of CD4-dependent Envs in the brain of HIV-1 clade B^+^ subjects described here. Why do HIV-1 Envs evolve CD4 independence in the context of SHIV and macaques but remain CD4 dependent with an enhanced Env:CD4 affinity in the context of HIV-1 and humans? One possibility is that the macaque models of SIV or SHIV neurological disease occur in animals that are immunosuppressed or are rapid progressor animals where neurological disease progresses before a potent immune response can be elicited. For example, the neuroAIDS model used by Cheng-Mayer’s group occurred in rapid-progressor macaques with poor antibody responses [62]. SIVmac models of neurological disease also often require immune modulation, e.g., CD8 cell depletion [4, 59]. These conditions may favor the evolution of variants with more open Env trimers that are capable of interacting directly with coreceptors. Such variants are likely to be highly neutralization sensitive [55] and potentially selected against in HIV-1-infected humans, where the neutralizing antibody response is reasonably robust [32, 33].

It should also be noted that it was necessary for pathogenic SHIVs to go through serial passage in macaques to select for neuropathogenic viral forms [4, 59, 62]. This adaptation results in the selection of variants that are able to efficiently use macaque CD4, where changes in the trimeric Env quartenary structure have been described [5]. These (and other changes) may predispose SHIV Envs to evolve CD4 independence.

CD4-independent infection of CCR5^+^ cells may involve variants that have evolved a higher Env:CCR5 affinity. We evaluated whether brain or mac-tropic HIV-1 Envs have a higher affinity for CCR5 that predisposes them to CD4 independence even though they were CD4-dependent. Several previous studies have indicated that enhanced or altered Env:CCR5 interactions are associated with HIV-1 R5 macrophage tropism [25, 46, 48, 51]. For example, Salemi et al. reported that mac-tropic Envs were more dependent on determinants in the CCR5 N-terminus than non-mac-tropic Envs [48]. The roles of different CCR5 residues or domains in Env binding were not tested here. However, we did previously report that R5 Env sensitivity to CCR5 antagonists was variable but did not correlate with macrophage tropism [37, 41]. Here, we show that R5 gp120s vary in their ability to bind CCR5, with the non-mac-tropic gp120s conferring the most efficient binding, while several gp120s from highly mac-tropic Envs bound less efficiently. These data are not inconsistent with differences in the interactions of Env with the N-terminus of CCR5 as reported by Salimi et al. [48]. However, they do not indicate that mac-tropic R5 Envs consistently evolve an enhanced or highly efficient CCR5 interaction to facilitate macrophage infection.

It was not possible to establish an Env:CCR5 binding assay using trimeric Envs expressed on 293T cells, since there are no suitable forms of soluble CCR5 to use in such an assay. Highly purified forms of CCR5 have been used to produce high-resolution structures of CCR5 [54], but these contain CCR5 complexed with maraviroc, a CCR5 antagonist that prevents Env:CCR5 binding. It is also important to note that soluble gp120 produced from 293T cells may contain unnatural dimers, which have been reported to affect estimates of gp120:CD4 affinity, although effects on Env:CCR5 interactions have not been reported [10]. The gp120 preparations used here contained small amounts of dimers, although some had somewhat larger amounts (not shown). The presence of dimers in the gp120 preparation did not correlate with the extent of CCR5 binding, mac-tropism or tissue origin of Envs. However, we cannot state for certain that the presence of dimers in some gp120 preparations did not have some influence on gp120:CCR5 binding.

Previous studies by our group and others have found a strong compartmentalization of highly mac-tropic R5 HIV variants in brain tissue in individuals with neuroAIDS. In contrast, only a few mac-tropic Envs were detected in brain tissue of patients without neurological complications but formed phylogenetic clusters that were distinct from those of most non-mac-tropic Envs. We have also reported that highly mac-tropic gp120 variants carrying a lower charge were nearly universal in the brain when compared to Envs from immune tissue of neuroAIDS patients. However, only one patient with normal neurology carried brain Envs with a significantly lower charge than those in immune tissue, and it is possible that low Env charge is associated with the development of neurovirulence. A relationship between increased neuropathology and CD4 independence in an SHIV-induced encephalitis model has been described previously [62]. However, the correlation between the overall positive charge and variation of CD4 independence in SHIV Envs has not yet been studied.

In summary, in this study we investigated a diverse HIV-1 clade B Env population to assess whether highly mac-tropic HIV-1 Envs derived from brain tissue have evolved an enhanced affinity for CCR5 and/or are able to infect cells without CD4. No evidence of such CD4 independence was detected. We conclude that mac-tropism in SIV and SHIV models of neurotropism evolves via mechanisms that differ from those of HIV-1 clade B in humans.
